# Formation of Biofilm by *Tetragenococcus halophilus* Benefited Stress Tolerance and Anti-biofilm Activity Against *S. aureus* and *S. Typhimurium*

**DOI:** 10.3389/fmicb.2022.819302

**Published:** 2022-03-01

**Authors:** Shangjie Yao, Liying Hao, Rongqing Zhou, Yao Jin, Jun Huang, Chongde Wu

**Affiliations:** ^1^College of Biomass Science and Engineering, Sichuan University, Chengdu, China; ^2^Key Laboratory of Leather Chemistry and Engineering, Ministry of Education, Sichuan University, Chengdu, China; ^3^State Key Laboratory of Oral Diseases, West China Hospital of Stomatology, Sichuan University, Chengdu, China

**Keywords:** *Tetragenococcus halophilus*, biofilm formation, environmental stress, biofilm matrix, aggregation activity, anti-biofilm activity

## Abstract

*Tetragenococcus halophilus*, a halophilic lactic acid bacterium (LAB), plays an important role in the production of high-salt fermented foods. Generally, formation of biofilm benefits the fitness of cells when faced with competitive and increasingly hostile fermented environments. In this work, the biofilm-forming capacity of *T. halophilus* was investigated. The results showed that the optimal conditions for biofilm formation by *T. halophilus* were at 3–9% salt content, 0–6% ethanol content, pH 7.0, 30°C, and on the surface of stainless steel. Confocal laser scanning microscopy (CLSM) analysis presented a dense and flat biofilm with a thickness of about 24 μm, and higher amounts of live cells were located near the surface of biofilm and more dead cells located at the bottom. Proteins, polysaccharides, extracellular-DNA (eDNA), and humic-like substances were all proved to take part in biofilm formation. Higher basic surface charge, greater hydrophilicity, and lower intracellular lactate dehydrogenase (LDH) activities were detected in *T. halophilus* grown in biofilms. Atomic force microscopy (AFM) imaging revealed that biofilm cultures of *T. halophilus* had stronger surface adhesion forces than planktonic cells. Cells in biofilm exhibited higher cell viability under acid stress, ethanol stress, heat stress, and oxidative stress. In addition, *T. halophilus* biofilms exhibited aggregation activity and anti-biofilm activity against *Staphylococcus aureus* and *Salmonella Typhimurium*. Results presented in the study may contribute to enhancing stress tolerance of *T. halophilus* and utilize their antagonistic activities against foodborne pathogens during the production of fermented foods.

## Introduction

Biofilms are aggregates consisting of cells and the biofilm matrix, and for the majority of microbes in nature, biofilm formation on a surface is an instinctive and survival behavior (Costerton et al., 1999; Parsek and Singh, 2003). As is known, biofilm is involved in biofilm-associated infections and inherent antibiotic resistance in modern medicine (Ribeiro et al., 2016). Meanwhile, some microorganisms such as *Bacillus* spp. caused serious hygiene problems and economic losses in the food industry because of the formation and release of spores in biofilms and no infallible strategy can be used to eliminate biofilms (Faille et al., 2014). Apart from these harmful effects, biofilms also exhibited beneficial performances. For instance, they play positive roles in bioremediation processes, toxic effluent treatment, reduction of ammonia and nitrate concentrations, and antimicrobial compound production (Schlegelová and Karpísková, 2007). The greater tendency to adhere on surfaces during biofilm formation benefits biomass separation in the production of alcoholic beverages (Speranza et al., 2020).

In addition, previous research demonstrated that formation of biofilm conferred cells higher resistance and probiotic properties to environmental stresses (Chamignon et al., 2020). As is known, microorganisms would encounter various environmental stresses such as high salt stress, acid stress, extreme temperature, ethanol stress, and oxidative stress during the production of fermented foods. Biofilms can provide a barrier between cells and adverse environment factors. In the winemaking process, biofilms increased the ethanol tolerance of *Oenococcus oeni* during the alcoholic fermentation, and the microorganisms played an effective functional performance in the following malolactic fermentation (Bastard et al., 2016). The regulation of biofilm formation to enhance stress tolerance of cells has been proved to be feasible and effective. The addition of potassium ions contributed to biofilm formation by *Lactobacillus plantarum* through regulating the expression of multiple genes, and the freeze-drying survival rate of cells improved (Jingjing et al., 2021). What is more, biofilm dispersal after biofilm maturation can provide planktonic cells and increase the functional microbial cell amount. Some reports introduced the biotechnological application of controlled biofilms formed by beneficial microbes in fermentation mainly involved in control on food spoilage or poisoning microorganisms in biofilms and fermentation optimization, as well as improvement of yield and quality of food fermentations (Berlanga and Guerrero, 2016).

In recent years, more and more studies on the biofilm-forming abilities of lactic acid bacteria (LAB) have been published. Kubota et al. (2008) investigated the biofilm formation of *L. plantarum*, *Lactobacillus brevis*, and *Lactobacillus fructivorans*, and the biofilm cells had longer length and higher resistance to acetic acid and ethanol than the planktonic cells. Akoğlu (2020) explored environmental conditions for biofilm formation by six LAB strains from a local cheese in Turkey, and the results showed that acidic pH, increase in glucose and lactose concentrations, and decreasing salt concentration benefited the biofilm formation of *Enterococcus lactis* EC61 and *Enterococcus faecali*s EC41. Further, the ability of LAB biofilm to inhibit foodborne pathogens was investigated, and *Pediococcus pentosaceus* and *Enterococcus faecium* could form biofilms which showed antimicrobial activities against *Bacillus cereus*, *Escherichia coli*, and *Salmonella enterica* (Tatsaporn and Kornkanok, 2020).

*Tetragenococcus halophilus* widely exists and is usually a starter culture added in salty fermented foods, contributing to flavor formation, and development in these processes (Jeong et al., 2017; Udomsil et al., 2017; Harada et al., 2018). This study aimed to investigate the formation of biofilm by *T. halophilus*, and the properties between the biofilms and planktonic cells were compared. Further, the effect of biofilm formation on stress tolerance, aggregation activity, and anti-biofilm activity against some model foodborne pathogens of *T. halophilus* was explored. Results presented in this study may contribute to further understanding the formation of biofilm by *T. halophilus*, and prevention and control of bacterial biofilm in food fermentation.

## Materials and Methods

### Strains, Culture Conditions, and Biofilm Formation

The strain used in this study was *T. halophilus* CGMCC 3792. *T. halophilus* was isolated from soya sauce moromi and identified *via* physiological, biochemical, and 16S rDNA sequence analyses and stored at the China General Microbiological Culture Collection Center (CGMCC) (Wu et al., 2013).

*Tetragenococcus halophilus* cultures stored at −80°C were inoculated into MRS (de Man, Rogosa, and Sharp) medium (Oxoid, Hampshire, United Kingdom) and were incubated at 30°C for 24 h. Then the cell suspension was inoculated with an inoculum size of 1% (v/v) into 100 ml fresh MRS medium containing 6% NaCl, unless explicitly stated. The diluted cultures with a final concentration of 1 × 10^5^ CFU/ml were dispensed into a conical flask or a 96-well or 24-well microtiter polystyrene plate (Sangon Biotech, Shanghai, China). Biofilm formation was performed statically at 30°C. For planktonic cell culture, the MRS medium containing a final *T. halophilus* cell concentration of 1 × 10^5^ CFU/ml was incubated in a shaker at 100 rpm and 30°C.

In order to investigate the effects of salt content [0, 3, 6, 9, and 12% (m/v)], ethanol content [0, 2, 4, 6, 8, and 10% (m/v)], pH (6.5, 5.8, 5.0, 4.6, 4.2, and 3.8) in medium, sugar addition (glucose, galactose, lactose, sucrose, and maltose), culture temperature (20, 25, 30, 37, and 42°C), and support materials (polystyrene, glass, and 304 stainless steel with 2B surface finish) on the formation of biofilm, cells were incubated at 30°C for 72 h. As for glass and stainless steel coupons, they were washed with detergent and then sterilized by autoclaving.

The pathogenic bacteria used for the determination of co-aggregation activity and anti-biofilm activity were purchased from ATCC. *Staphylococcus aureus* ATCC 6538, *Salmonella Typhimurium* ATCC 14028, and *Listeria monocytogenes* ATCC 19115 were all grown in tryptic soy broth (TSA, Oxoid, United Kingdom) statically at 37°C.

### Crystal Violet Assay

Crystal violet assay was used for biofilm quantification. The non-adherent cells were removed by dipping each sample in water. Then wells were dried at 35°C for 4 h. Biofilms were stained with 1% (w/v) crystal violet for 15 min. After that, the wells were washed thoroughly with water and dried at 35°C for 4 h. The retained crystal violet was dissolved in ethanol-acetone (80:20, v/v) with an equal volume of cell suspension. After dissolution overnight, this solution was transferred to a new microplate and the absorbance at 492 nm was measured by a microplate reader (HIMF, BioTek, Winooski, VT, United States).

### Scanning Electron Microscopy Analysis

As for scanning electron microscopy (SEM) analysis, the biofilms were incubated in 24-well plates with a sterile 12-mm-diameter glass coverslip in the bottom. After culture, the biofilms were washed as above, then the biofilms and planktonic cells were fixed with 2.5% glutaraldehyde overnight. Dehydration was processed in graded ethanol solutions (30, 40, 50, 60, 70, 80, 85, 90, 95, and 100%) for 15 min each. After drying, the biofilms were scraped off with a knife from glass coverslips. Both biofilms and planktonic cells were sputter-coated with gold and observed by a scanning electron microscope (Apreo S, Thermo Fisher Scientific, Waltham, MA, United States). The scanning parameter was set at 15.00 kV.

### Confocal Laser Scanning Microscopy Analysis

The biofilm was incubated in a 15-mm-diameter cell culture dish with a glass bottom (NEST Biotechnology, Wuxi, China). The supernatant was removed, and biofilm was rinsed once in 0.9% saline solution, then fixed in 0.5 mM SYTO 9 Green fluorescent nucleic acid stain (Invitrogen, Carlsbad, CA, United States) and 100 μg/ml propidium iodide (PI) for 30 min. Then, the cell culture dish was examined using an Olympus FluoView FV3000 CLSM (Olympus Corporation, Tokyo, Japan) under a ×40 oil immersion objective lens. The 3D architecture of biofilm was scanned with a z-direction of 0.4 μm between each xy image.

### Analysis of the Composition of the *T. halophilus* Biofilm Matrix

To investigate the composition of the *T. halophilus* biofilm matrix, proteinase, DNase, and sodium periodate were used in dissociation of biofilms (Thibeaux et al., 2020). After 48 h of incubation, 10 μl proteinase K (1 mg/ml in 0.1 M Tris, 0.5% SDS, Roche Diagnostics GmbH, Mannheim, Germany), 10 μl DNase I (1 mg/ml in 0.1 M Tris, 1 mM DTT, Roche Diagnostics GmbH, Germany), and 10 μl sodium periodate (100 mM in 0.1 M Tris, Sangon Biotech, China) were added directly to the biofilms. Then, 10 μl of 0.1 M Tris was added as control. Then, the 96-well plate was incubated at 30°C for 24 h. Then, biofilms were quantified by crystal violet assay.

In addition, the main components of the *T. halophilus* biofilm matrix were analyzed by three-dimensional fluorescence spectrum (3D-EEM). The biofilms incubated for 72 h were collected and washed twice by centrifugation at 8,000 *g* for 5 min. Deionized water was added into the centrifuge tubes containing biofilms and vortex-mixed for 10 min to release the biofilm cells. After standing for 2 min, the settle at the bottom was removed. Then, the OD_600_ of the suspension was adjusted to 0.5 with water. Cation exchange resin (Sangon Biotech, China) with a mass of 70 g/g dry cell was added in the suspension, and the suspension was placed in a shaker (200 rpm, 4°C) for 6 h. After centrifugation at 10,000 *g* for 10 min, the supernatant was collected and filtered with a 0.22-μm sterile filter. The same treatment was done for planktonic cells incubated at 100 rpm for 72 h. The EEM spectra of the biofilm matrix were measured using a luminescence spectrometer (F7100, Hitachi, Tokyo, Japan). The emission spectra were collected from 250 to 550 nm at 10-nm increments by varying the excitation wavelength from 200 to 500 nm at 10-nm increments. Excitation and emission slits were maintained at 5 nm. The scanning speed was set at 1,200 nm/min for all the measurements (Li et al., 2020). The software Origin 2019 (OriginLab Inc., Hampton, VA, United States) was used to analyze the EEM data.

### Surface Charge, Contact Angle, Zeta Potential, and Lactate Dehydrogenase Activity Measurement

The biofilm and planktonic cells were incubated as the method described above in 3D-EEM analysis. Both cells were collected and washed twice by centrifugation at 8,000 *g* for 5 min and resuspended in PBS (pH 7.0). For surface charge, basic and acidic surface characteristics were measured by using chloroform, a Lewis acid, and ethyl acetate, a Lewis base according to the method described by Colloca et al. (2000).

As for contact angle measurement, the biofilm and planktonic cell suspension with an OD_600_ of 1.0 was prepared as the method described in 3D-EEM analysis. The cells in 200 ml suspension were deposited on 0.45-μm pore-size HA membrane filters (Jinteng, Tianjin, China), and then the filters were dried until reaching constantly to remove free but bound water on cell surfaces. The transient contact angles of cell surfaces were recorded at 0.2 s after the water touching on *T. halophilus* lawns using a contact angle meter (SPCAX1, HARKE, China) under temperature 20 ± 2°C, humidity 60 ± 5%. For the zeta potential measurement, cells were suspended in 10 mM PBS (pH 7.0) to obtain an OD600 of 0.8. The zeta potential of biofilms and planktonic cells was determined by using a Nano ZSP model zetasizer instrument (ZEM5600, Malvern Instruments Ltd., United Kingdom) with disposable DTS1070 electrophoresis cuvettes, and the sample was analyzed in sextuplicate.

To determine the lactate dehydrogenase (LDH) activities, the cells were disrupted by using an ultrasonic cell disruptor (JY92-11 N, SCIENTZ, Ningbo, China) with a Φ2 amplitude transformer for 25 min (ultrasound for 4 s with a 1-s interval) in an ice bath to obtain a cell homogenate. The cell homogenate was treated according to procedures of the LDH activity assay kit (Nanjing Jiancheng Bioengineering Institute, Nanjing, China). The samples were monitored by a spectrophotometer (L7, INESA Instrument, Shanghai, China) at 636 nm. Protein content was measured by the Coomassie brilliant blue method (Kruger, 1994), and the activity of LDH was expressed as U/gprot.

### Adhesion Force Analysis by Atomic Force Microscopy

Adhesion force measurements were carried out by using atomic force microscopy (AFM). The biofilm and planktonic cells were incubated in a 24-well plate containing sterile 12-mm-diameter glass coverslips for 72 h. The supernatant was removed, and biofilm was rinsed once in 0.9% saline solution, then dried under natural conditions (temperature 20–25°C, humidity 30–40%). The planktonic cells were washed once after centrifugation at 8,000 *g* for 10 min and resuspended in 0.5 ml 0.9% saline solution. Then, 10 μl cell suspension was uniformly smeared onto glass coverslips and dried under natural conditions. Adhesion force on the surface of both cells was analyzed by using a SPM-9700 AFM (Shimadzu, Kyoto, Japan) with a CSG 10 Au cantilever (TipsNano, Tallinn, Estonia) of which force constant was 0.11 N/m. Images of 256 × 256 pixels and force curves were collected at a scanning rate of 1 Hz in Constant Force mode.

### Cell Viability Assessment During Environment Stresses

Biofilms were incubated statically in a 24-well plate, and planktonic cells were incubated in conical flasks at 100 rpm. The biofilms were divided into two parts. A part of biofilm cells which served as biofilm-dispersed cells grown to the mid-logarithmic phase was harvested and washed once by centrifugation at 8,000 *g* for 10 min, then vortex-mixed for 10 min to release the biofilm cells. Planktonic and biofilm-dispersed cells were resuspended in fresh MRS medium adjusted to pH 4.15 with hydrochloric acid or fresh MRS medium with or without 12% (v/v) ethanol or 0.075% (v/v) H_2_O_2_. For another part of biofilms, the supernatant was removed and biofilms were rinsed once in 0.9% saline solution. After removing saline solution, the same medium described above was added in plates gently. Planktonic and biofilm-dispersed cells were stressed at 30°C and 100 rpm, while biofilm cells were stressed statically at 30°C. Heat stress was performed in a 52°C bath for 1.5 h. Acid stress and oxidative stress were performed for 1.5 h, ethanol stress for 3 h. The live cell count method and survival rate calculation were described as in our previous study (Yao et al., 2021).

### Aggregation Activity and Anti-biofilm Activity

Aggregation activity and anti-biofilm activity against some pathogens of *T. halophilus* biofilms were determined. For aggregative abilities, auto-aggregation of *T. halophilus* planktonic and biofilm cells and their co-aggregation abilities with pathogenic bacteria were evaluated as described by Campana et al. (2017).

The capability of *T. halophilus* biofilm to inhibit the biofilm formation by pathogenic bacteria was determined according to a previous method (Tarrah et al., 2019) with minor modifications. Briefly, after incubation for 72 h, the broths were carefully discarded and the *T. halophilus* biofilm on the bottom of the 24-well plate was washed with PBS (pH 7.0) to remove non-adherent cells. Then, the wells were inoculated with cell suspensions of *S. aureus*, *S. Typhimurium*, or *L. monocytogenes* containing 10^5^ CFU/ml in TSA broth and cells were incubated at 37°C for a further 48 h. After discarding the broth and washing the biofilm, pathogenic bacteria were counted by using Baird–Parker medium for *S. aureus*, chromogenic *Salmonella* medium for *S. Typhimurium*, and PALCAM medium for *L. monocytogenes*.

### Statistical Analysis

All analyses were conducted in triplicate. Significant differences were tested by one-way analysis of variance (ANOVA) using IBM SPSS Statistics Software (version 22) at *p* < 0.05, and Tukey’s test was applied for comparison of means.

## Results and Discussion

### Biofilm Formation by *T. halophilus* Across Diverse Conditions

**FIGURE 1:**
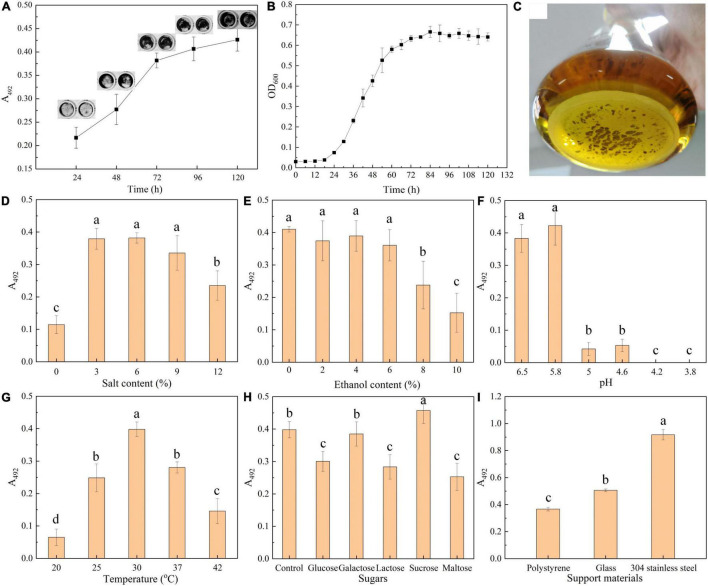
Biofilm formation by *T. halophilus*. **(A)** Time course of biofilm formation quantified by crystal violet assay. Insets show images of stained biofilm in gray. **(B)** Growth curves of *T. halophilus* under stationary culture conditions. **(C)** Picture of *T. halophilus* biofilm taken after 120 h of incubation. **(D–G)** Biofilm incubated for 72 h in MRS with different salt contents **(D)**, ethanol contents **(E)**, pHs **(F)**, and temperatures **(G)**. The pH value of the MRS medium was 5.8 after autoclaving without any adjustment. **(H)** Biofilm incubated for 72 h in MRS added with extra 5 g/l glucose, galactose, lactose, sucrose, or maltose. **(I)** Biofilm incubated for 72 h in a 24-well plate containing a piece of polystyrene, glass, or 304 stainless steel (12-mm-diameter). Columns labeled with different lowercase letters are significantly different at *p* < 0.05.

We firstly explore the relationship between biofilm formation and *T. halophilus* cell growth. The result of crystal violet assay (Figure 1A) showed that the biofilm grew over time after inoculation, and there was a rapid increase in biofilm from 24 to 72 h. The insets in Figure 1A show a consistent growth of the area of biofilm on the bottom of a 96-well plate. As shown in the growth curves in Figure 1B, *T. halophilus* reached its logarithmic phase and stationary phase after 24 and 72 h of culture, respectively. It was obvious that the biofilm amount was positively correlated with cell density. In the stationary phase, a slight increase in biofilm mass was observed when cell biomass was kept constant, and this may be attributed to the accumulation of the biofilm matrix due to cell secretion and death (Desai et al., 2019). After incubation for 120 h, there was a thick biofilm in the bottom of the conical flask (Figure 1C).

Previous report suggested that environmental conditions were closely reported to the formation and architecture of biofilm (Campoccia et al., 2013; Zhang et al., 2021). In this section, effects of environmental conditions including salt concentration, ethanol concentration, pH, sugar addition, temperature, and support materials on biofilm formation were investigated. *T. halophilus* is a halophilic LAB that can tolerate up to 25% w/v NaCl and optimal growth at 5–10% NaCl (Taniguchi et al., 1988). As shown in Figure 1D, when the salt content increased to 3, 6, or 9%, there was an obvious biofilm formation. A suitable concentration of salt was favorable for *T. halophilus* survival and proliferation. In a research of *Vibrio fischeri* biofilm formation (Marsden et al., 2017), salt availability was found to contribute to the upregulated expression of the *syp* locus which encodes some proteins predicted to produce and export a polysaccharide component of the biofilm matrix.

The effect of ethanol on biofilm formation was investigated (Figure 1E), and the results showed that biofilm formation was significantly reduced when the concentration of ethanol exceeded 8%. Ethanol has no significant effect on biofilm formation until the content exceeded 8%. In previous reports, ethanol around 2.5–3.5% initiated a stronger biofilm formation by *S. aureus* (Tango et al., 2018), while the induction of ethanol with low content on biofilm formation was not observed in this study. Thus, whether ethanol could promote biofilm formation depended on the species of the microorganism.

As shown in Figure 1F, the acid environment can effectively inhibit biofilm formation of *T. halophilus*. There was a great decrease in biofilm mass when the pH was lower than 5.0, and no biofilm was detected when the pH was under 4.2. In contrast, Dimakopoulou-Papazoglou et al. (2016) reported that the formation of biofilm by *Salmonella enterica* was observed at a pH lower than 3.8. At acidic conditions, protonation of the biofilm matrix resulted in changes in charge and morphological characteristics of the biofilm matrix, thereby influencing the aggregation of biofilm (Teng et al., 2016; di Biase et al., 2020). In addition, Wang et al. (2016) reported that acid stress reduced biofilm formation by decreasing the amounts of various components including polysaccharides and proteins in the biofilm matrix.

In Figure 1G, biofilm amount showed a peak distribution with culture temperature, and the optimal temperature required for biofilm formation was 30°C. *T. halophilus* exhibited the best growth performance when the temperature was 30°C (Wu et al., 2013), and the highest biofilm amount was formed when the culture temperature was 30°C. Similar results were also reported by Pumeesat et al. (2017) which suggested that temperature significantly influenced the bacterial biofilm formation.

Figure 1H displays the effect of sugar addition on biofilm formation, and the results suggested that all the supplemented sugars (glucose, lactose, and maltose) except galactose and sucrose inhibited the biofilm formation. Sucrose exhibited an ability to enhance biofilm formation by *T. halophilus*. In the study of Slížová et al. (2015), they observed a highly positive biofilm formation in the growth medium added with 1% glucose, 1% galactose, and 1% lactose, respectively, while a gradual increase in the sugar concentration led to a significant decrease in biofilm formation. In specific intercellular adhesion, the lectin–glycan receptor interaction would be inhibited by carbohydrate or glycoconjugate (León-Romero et al., 2016). Cai et al. (2016) suggested that strong biofilms formed by *Streptococcus mutans* in culture medium contain a wide range of sucrose from 0.5 to 20%. It was reported that the carbohydrates in medium regulated the biosynthetic pathway and the sugar composition of exopolysaccharides from *Lactobacillus* (Welman and Maddox, 2003). Moreover, sucrose has proven its ability to change the extracellular composition of the biofilm, increasing bacterial adhesion and biofilm accumulation by several *Streptococcus* species (Souza et al., 2019). In a proteomic study, sucrose was found to induce the differential expression of several enzymes in *Lactobacillus sakei*, among which higher levels of dextransucrases help *L. sakei* using sucrose as substrate for biosynthesis of high molecular weight dextran which was responsible for biofilm formation in diverse LAB species (Prechtl et al., 2018). In addition, a sucrose-dependent adhesion which required sucrose for binding was reported in some studies, and in the process, glucosyltransferases (Gtfs) and the glucan-binding proteins (Gbps) were regarded as sucrose-dependent virulence factors (Mieher et al., 2018).

To test biofilm formation on different support materials, three kinds of support surfaces (polystyrene, glass, and stainless steel) were employed to evaluate the effect of support materials on biofilm formation. The results showed that *T. halophilus* was able to form biofilm on each of the surfaces, and the strongest biofilm was formed on the surface of stainless steel, followed by glass (Figure 1I). Di Bonaventura et al. (2008) reported that comparable amounts of biofilm were formed by *L. monocytogenes* on both stainless steel and glass at 37°C compared with polystyrene, and biofilm levels were highest on glass when incubated below 37°C. They concluded that temperature probably modified the cell surface properties such as hydrophobicity and attachment factors, leading to the differences in biofilm levels on polystyrene, glass, and stainless steel at different temperatures. Similarly, Bonsaglia et al. (2014) investigated the abilities of 32 strains of *L. monocytogenes* to form biofilms on three different surfaces (polystyrene, glass, and stainless steel). According to these authors, the bacteria adhered better to hydrophilic surfaces (stainless steel and glass) than to hydrophobic ones (polystyrene). In addition, an increase in iron availability might also contribute to biofilm formation and maturation (Hayrapetyan et al., 2015). Ceramic cylinders and stainless steel tanks are usually used as the containers of traditional fermented foods. It is necessary to assess the adhesion and biofilm-forming propensity of *T. halophilus* on stainless steel and ceramic coupons in further study.

### Scanning Electron Microscopy Images of the Biofilm Matrix

**FIGURE 2:**
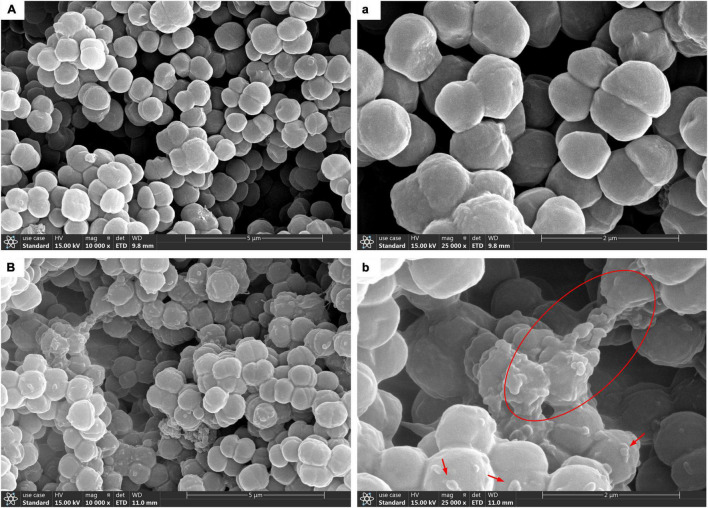
Scanning electron microscopy (SEM) images of *T. halophilus* biofilms. **(A,a)** SEM images of planktonic cells taken at ×10,000, ×25,000 magnification, respectively. **(B,b)** SEM images of biofilms taken at ×10,000, ×25,000 magnification, respectively. The red circle displays the network-like structures, and arrows were surface attachments.

Scanning electron microscopy technology was used for further morphological observation of biofilm. The SEM micrograph in Figures 2A,a showed that planktonic *T. halophilus* cells independently existed in the form of dyad or tetrad, and the cell surface was free of attachments. In biofilm, the cells adhered with each other by a layer adhesive substance on the cell surface Figures 2B,b. In Figure 2b, it can be observed clearly that the surface was attached with lump-like structures and some network-like structures bridged cells. The similar structures were found in biofilm by *S. mutans* (Zhang et al., 2020). The better adhesive properties of biofilm than planktonic cells might be explained by the layer of biofilm matrix coating the biofilm cells (Volle et al., 2008).

### 3D Architecture and Cell Distribution of *T. halophilus* Biofilm

**FIGURE 3:**
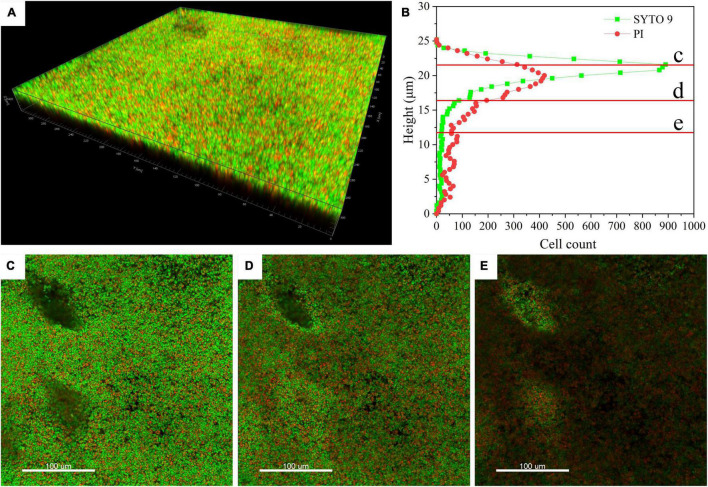
Confocal laser scanning microscopy (CLSM) images of *T. halophilus* biofilms. **(A)** CLSM images of biofilm stained by PI (dead cells, red) and SYTO 9 (live cells, green). **(B)** The distribution of live cells and dead cells at different heights. **(C–E)** CLSM images at different focal planes which are marked c, d, and e in figure B, respectively. The three-dimensional reconstruction and cell distribution were performed by Imaris 7.4.2.

Confocal laser scanning microscopy (CLSM) analysis can provide information about cell localization in biofilm and visualize other matrix components by specific cell fluorescent probes or specific stains. For CLSM analysis, *T. halophilus* biofilms were stained by SYTO 9 and PI, and the spatial organization of the biofilms by *T. halophilus* was visualized. Figure 3A shows that *T. halophilus* formed a dense biofilm that covered the entire surface. Figure 3B reveals the distribution of live and dead cells at different heights in biofilm with a thickness of at least 12 μm. From the surface to 5 μm deep, the amounts of live cells were more than those of dead cells, and higher amounts of dead cells were located under the height of 19.5 μm. The same result was clearly observed in Figures 3C–E. This phenomenon may be ascribed to the fact that many cells attached to the surface of the dish in the early stages of biofilm formation died and disintegrated, and the cells near the surface multiplied rapidly due to exposure to more nutrients. Gradient reduction of nutrients caused by nutrient consumption by cells and diffusion limitation, as well as accumulation of harmful metabolites including acids, was unfavorable to cell survival (Stewart and Franklin, 2008). The CLSM image of the lower layer within biofilms has lower fluorescence intensity. This phenomenon might be ascribed to the uneven distribution of stains in biofilms due to the densification of the biofilms.

### The Composition of Biofilm by *T. halophilus*

**FIGURE 4:**
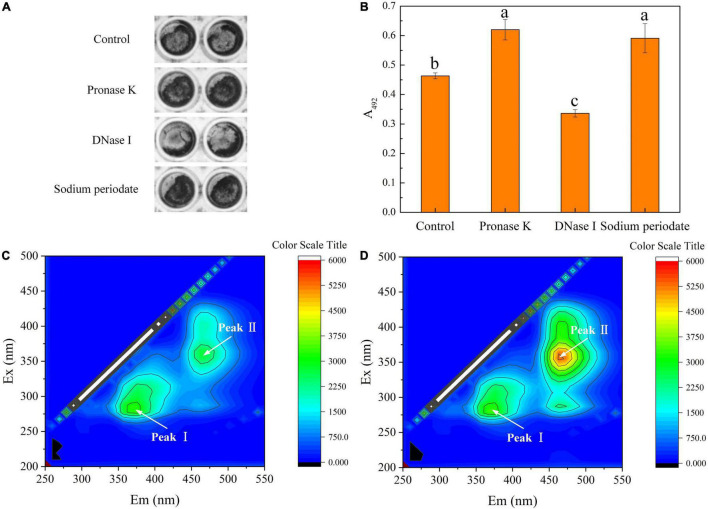
Composition of biofilm matrix. **(A)** Crystal violet staining of biofilms untreated or treated for 24 h with proteinase K, DNase I, and sodium metaperiodate. **(B)** Quantification of biofilms untreated or treated for 24 h with proteinase K, DNase I, and sodium metaperiodate. **(C)** EEM fluorescence spectra of the matrix from planktonic cells. **(D)** EEM fluorescence spectra of the biofilm matrix. Significantly different (*p* < 0.05) values are marked with different lowercase letters. The *T. halophilus* cells were incubated in 96-well plates for 48 h and then treated with proteinase K, DNase I, and sodium metaperiodate for 24 h.

The composition of the biofilm matrix was explored by using enzymatic or chemical treatments (Figures 4A,B). The images (Figure 4A) showed darker-colored and wider-area biofilms treated by proteinase K or sodium periodate than control. Further, after exposure to proteinase K or sodium periodate for 24 h, the biofilm amount quantified by crystal violet assay was surprisingly higher than that under untreated conditions (Figure 4B). In contrast, the biofilm amount declined significantly after being treated with DNase I compared with the untreated samples. It indicated that proteins, polysaccharides, and eDNA all took part in biofilm formation. Thibeaux et al. (2020) used the Vybrant™ CFDA/SE Green Cell Tracer and SYPRO Ruby stain to label cells and proteins in biofilm by *Leptospira interrogans*, respectively. The result suggested that proteins mainly existed on the surface of cells and play roles in intercellular adhesion, and proteins were not the main component of the biofilm matrix. These proteins can serve as lectins mediating specific intercellular adhesion in that the interaction force is stronger several times than non-covalent biological bonds (Dufrêne and Viljoen, 2020). Hence, proteinase K treatment may hydrolyze the lectins, thus damaging intercellular adhesion but not completely disrupting the biofilm in our study that led to higher optical density in crystal violet assay (Figure 4A). As for polysaccharides, they played important roles in nonspecific intercellular adhesion due to the extensive existence of hydroxyl, carbonyl groups, mannose, and uronic acids and maintenance of biofilm structure (Ma et al., 2006). It can be explained that the treatment of *T. halophilus* biofilms with sodium periodate led to removal of the polysaccharides and exposure of cell surface which benefited the binding of crystal violet to the cell structures in the stain stage (Chaignon et al., 2007). Meanwhile, the polymeric chain of the polysaccharides was destroyed by sodium periodate that resulted in a damage in non-specific intercellular adhesion but not complete disruption to the biofilm (Xiong and Liu, 2013; Casillo et al., 2018). Similar results were found in the study of Sager et al. (2015), which demonstrated that polysaccharides masked the adhesive structures on bacterial surfaces. The removal of polysaccharides led to a better surface exposure of some proteins which were important in biofilm formation, resulting in an increased biofilm stability, especially in washing steps. The result in the study indicated that extracellular DNA (eDNA) may be a dominant component of the biofilm matrix and exposure to DNase I dissociated the biofilm structure. It was reported that eDNA released from cells during lysis or by active secretion was responsible for biofilm stabilization and cellular communication in the formation of a lattice-like structure (Gloag et al., 2013; Jakubovics et al., 2013). Enzymatic degradation of DNase I can cause collapse of the biofilm matrix and release microbial cells from biofilm (Izano et al., 2008; Kim et al., 2017).

Figures 4C,D display the 3D-EEM results of the biofilm matrix from planktonic cells and *T. halophilus* biofilm, respectively. Two peaks of EEM fluorescence spectra were observed in both planktonic cells and the biofilm matrix. Moreover, the peaks are located at Ex/Em (excitation/emission) wavelengths of 280/370 nm (peak I) and 360/470 nm (peak II), respectively. According to previous reports (Zheng et al., 2016; Gao et al., 2020), peak I and peak II were referred to as aromatic protein-like fluorescence and humic acid-like fluorescence, respectively. It meant that aromatic proteins and humic acid may be the compositions of the biofilm matrix. No significant difference in the fluorescence intensity at Peak I observed in planktonic cells and biofilm matrix suggested that biofilm formation did not change the amount of aromatic protein-like substances attached to cell surface. Moreover, it meant proteins mainly bound to the cell surface. A higher fluorescence intensity at Peak II in the biofilm matrix than that in planktonic cells showed that humic-like structure was one of the compositions of the *T. halophilus* biofilm matrix.

### Effect of Biofilm Formation on Cell Surface Properties and LDH Activities

**FIGURE 5:**
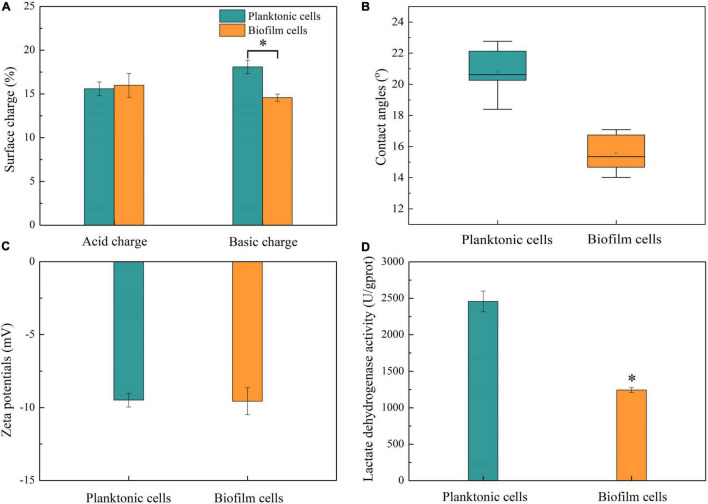
Cell surface properties and LDH activities. **(A)** Surface charges of planktonic and biofilm cells. **(B)** Contact angles of planktonic and biofilm cells. **(C)** Zeta potentials of planktonic and biofilm cells. **(D)** LDH activities of planktonic and biofilm cells. Asterisk indicates significantly different (*p* < 0.05).

The effects of biofilm formation on cell surface properties including surface charge, contact angles, zeta potentials, and intracellular enzyme activities were investigated (Figure 5). According to the result shown in Figure 5A, both the acidic and basic charges of biofilm and planktonic cells were lower than 35%; hence, the cells were classified as having low charged surfaces (Piwat et al., 2015). Moreover, there was a lack of predominant charge in cell surface due to similar acid and basic charges. Planktonic cells had a similar surface acid charge with biofilm cells and higher basic charge than the latter. The hydrophilicity of the biofilm and planktonic cells was determined by contact angle assay. Figure 5B shows that planktonic and biofilm cells can be wettable with contact angles <90°, and the hydrophilicity of biofilm cells was better than that of planktonic cells. The cell surface charge and hydrophobicity were related to the aggregation and adhesion of cells, which play important roles in initial attachment (Piwat et al., 2015). In this work, planktonic cells had more basic surface charge and stronger hydrophobicity than biofilm cells, implying that planktonic cells still had good auto-aggregating ability and adhesive properties. In addition, better hydrophilicity of biofilm cells might be attributed to the existence of hydrophilic polysaccharides in the biofilm matrix and coverage of hydrophobic membrane components by the biofilm matrix (Flemming and Wingender, 2010; Piwat et al., 2015; Al-Amshawee et al., 2021).

Zeta potential is considered as another important factor in microbial adhesion and reflected the microbial surface characteristics (Han et al., 2018). In this study, there was no statistical difference in zeta potential between biofilm and planktonic cells (Figure 5C).

Lactate dehydrogenase as a crucial enzyme is responsible for catalyzing the reversible reduction of pyruvate to lactate (Feldman-Salit et al., 2013). In this work, LDH activity was investigated to determine whether biofilm formation affected cell activity. LDH activities in biofilm and planktonic cells were analyzed, and the result is shown in Figure 5D. LDH activity in planktonic cells was about 2,450 U/gprot, which was about 2-fold higher than that in biofilm cells. In biofilm, cell viability was regulated due to the limitation of nutrient, accumulation of harmful metabolites, quorum sensing, and so on (Jefferson, 2004; Kalaiarasan et al., 2017). The cell density in biofilm was far higher than that in suspension culture, and a higher number of hypoactive and dead cells in biofilm were detected at the same concentration of protein.

### Effect of Biofilm Formation on Adhesion Force

**FIGURE 6:**
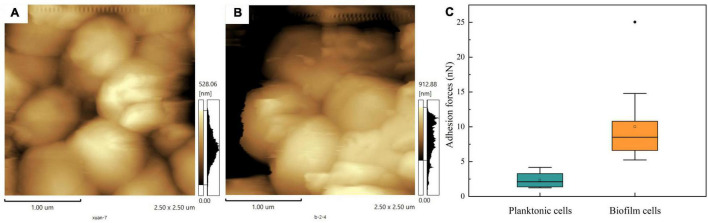
Cell surface adhesion forces. **(A)** AFM images of planktonic cells. **(B)** AFM images of biofilm. **(C)** Adhesion force on the surface of planktonic cells and biofilm.

Atomic force microscopy technology has been widely used to obtain images and direct physical information on the surface of cells. Using a cantilever to scan the surface of planktonic cells and biofilm, we harvested the height images of cells, shown in Figures 6A,B, respectively. Compared to planktonic cells, *T. halophilus* cells in biofilm exhibited a rough surface of the top layer in biofilms. During sample treatment, drying may change the physicochemical character of biofilms and planktonic cells. A comparison analysis of adhesion forces showed that surface adhesive forces of planktonic cells were lower than those of biofilm (Figure 6C), and biofilm had a wider distribution in surface adhesive forces. The existence of the biofilm matrix and surface attachments may contribute to the adhesive forces of biofilm cells and potential recruitment of planktonic cells (Flemming and Wingender, 2010; Dufrene and Persat, 2020).

### Effect of Biofilm Formation on Cell Viability During Environmental Stresses

**FIGURE 7:**
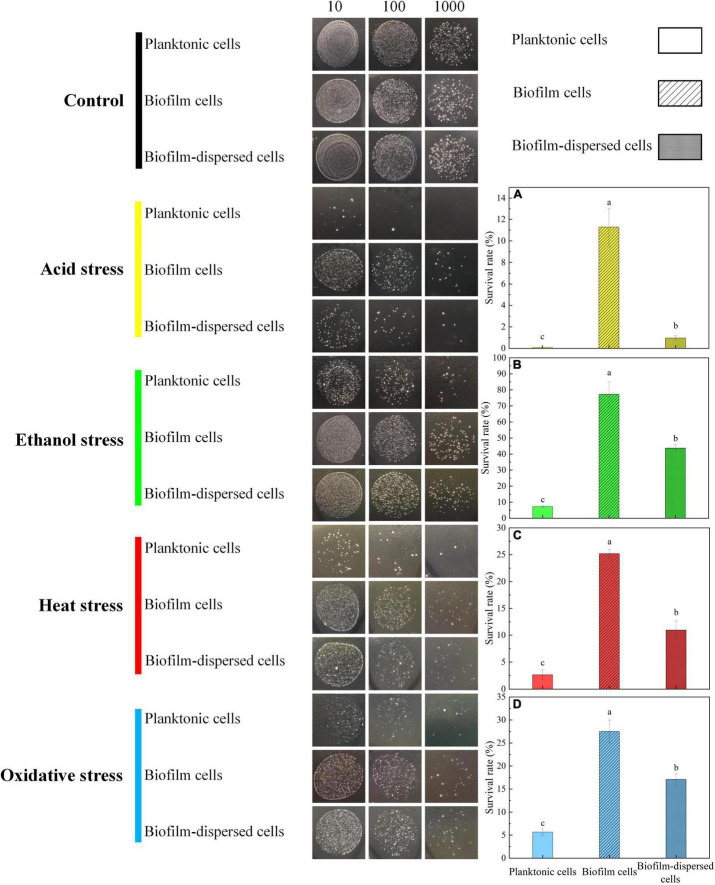
Viability of planktonic and biofilm cells after various environmental stresses. **(A)** The survival rate of *T. halophilus* cells after pH 4.15 acid stress for 1.5 h. **(B)** The survival rate of *T. halophilus* cells after 12% ethanol stress for 3 h. **(C)** The survival rate of *T. halophilus* cells after 52°C heat stress for 1.5 h. **(D)** The survival rate of *T. halophilus* cells after oxidative stress with 0.075% H_2_O_2_ for 1.5 h. The dilution factor for the live cell count method from left to right were 10, 100, and 1,000, respectively. Columns labeled with different lowercase letters are significantly different at *p* < 0.05.

Previous research showed that biofilm can protect microorganisms from extreme environmental stresses such as high temperature, extreme pH, high salinity, oxidative stress, antibiotics, and toxic metals (Booth et al., 2011; Gambino and Cappitelli, 2016; Wang et al., 2020). In the study, in order to investigate whether biofilm would benefit the cell viability of *T. halophilus* under environmental stresses, the survival rates of *T. halophilus* treated by acid stress, ethanol stress, heat stress, and oxidative stress were investigated (Figure 7). After exposure to acid stress (pH 4.15) for 1.5 h, a massive death occurred in both planktonic and biofilm cells. The survival rate of biofilm cells was 125-fold higher than that of planktonic cells. The cell viability of biofilm-dispersed cells was 10.7-fold higher than that of planktonic cells (Figure 7A). As for ethanol stress (12% ethanol), biofilm cells retained high viability after treatment for 3 h. The survival rate of biofilm-dispersed cells was 43.72% which was 6-fold higher than that of planktonic cells (Figure 7B). During temperature and oxidative stresses, about a quarter of biofilm cells survived when treated at 52°C and 0.075% H_2_O_2_ (Figures 7C,D). Moreover, the survival rate of biofilm cells was significantly higher than that of planktonic cells and biofilm-dispersed cells after heat stress and oxidative stress. It was worth noting that the biofilm-dispersed cells also displayed remarkably higher viability than that of planktonic cells during environmental stresses (Figure 7). An intact biofilm was crucial in the survival of biofilm cells when faced with environmental stresses. For biofilm-dispersed cells, the coverage of the biofilm matrix on the surface of cells may be one of the contributors to higher survival rates than those of planktonic cells. For some microorganisms, biofilm formation is one of the response mechanisms of cells to environmental stresses, and biofilm provides a natural barrier and protective layer to cells (Van Houdt and Michiels, 2010). During the biofilm formation, an increase in the expression of some genes or proteins might help biofilm cells gain stress tolerance capabilities (Philips et al., 2017). For instance, Bellenberg et al. (2019) demonstrated that a higher expression of 30 proteins involved in ROS degradation, thiol redox regulation, macromolecule repair mechanisms, biosynthesis of antioxidants, and metal and oxygen homeostasis was detected in pyrite-biofilm cells compared to that in planktonic cells, which helped protect cells against oxidative stress.

### Capability of *T. halophilus* on Aggregation and Inhibition to Biofilm Formation by Pathogenic Bacteria

**FIGURE 8:**
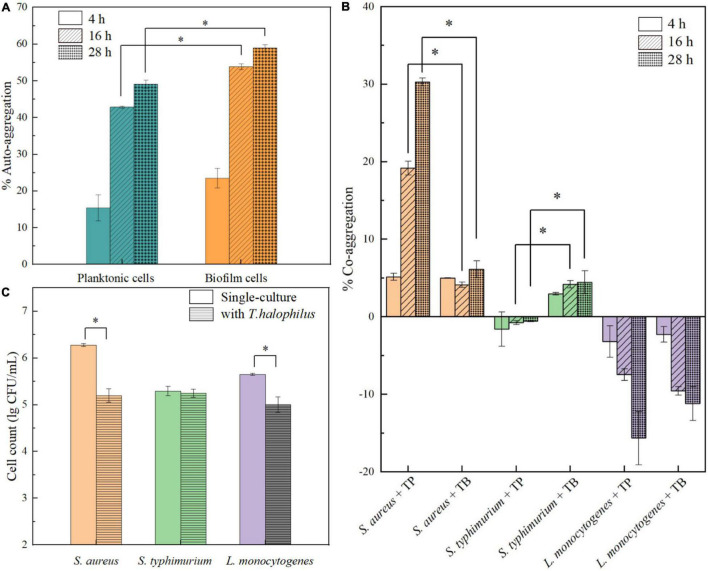
Anti-pathogenic activities of *T. halophilus* biofilm. **(A)** Auto-aggregation of *T. halophilus* planktonic and biofilm cells after 4, 16, and 28 h. **(B)** Co-aggregation of pathogenic strains with *T. halophilus* planktonic (TP) or biofilm cells (TB) after 4, 16, and 28 h. **(C)** Biofilm cell counts of pathogenic bacteria after incubation at 37°C for 48 h with *T. halophilu*s biofilms. Asterisk indicates significant difference (*p* < 0.05).

In the process of natural biofilm formation, microbial aggregation including auto-aggregation and co-aggregation plays a crucial role in pre-formation of cell clusters and adhesion (Kragh et al., 2016). It is well known that co-aggregation of probiotic bacteria can prevent the colonization of pathogenic species on the host or surfaces (García-Cayuela et al., 2014). In this study, the aggregation abilities of *T. halophilus* planktonic and biofilm cells were investigated. In Figure 8A, the auto-aggregation values of planktonic and biofilm cells both increased over time. The biofilm cells had higher percentages of aggregation (53.85 and 58.94%) than planktonic cells after 16 and 28 h, respectively. For co-aggregation abilities, the co-aggregation of *T. halophilus* with pathogenic species was strain-specific (Figure 8B). *T. halophilus* planktonic cells exhibited increased co-aggregation with *S. aureus* over time, and the biofilm cells had a low co-aggregation value with *S. aureus* (about 5%). There was no co-aggregation detected between *T. halophilus* planktonic cells and *S. Typhimurium*, while biofilm cells showed a level of co-aggregation with *S. Typhimurium* similar to *S. aureus*. Interestingly, the negative co-aggregation values between *T. halophilus* and *L. monocytogenes* meant that the mixing of the two strain cells inhibited the auto-aggregation of *T. halophilus* and/or *L. monocytogenes*. Figure 8C shows the biofilm inhibitory activities of *T. halophilus*, and the cell counts of *S. aureus* and *L. monocytogenes* in biofilms declined significantly in the presence of *T. halophilus* biofilms, but no obvious change in the cell counts of *S. Typhimurium* biofilm was observed. These results indicated that *T. halophilus* biofilms could reduce the formation of *S. aureus* biofilms and *L. monocytogenes* biofilms.

Auto-aggregation of *T. halophilus* observed in our study showed a strong trend in biofilm formation, and stronger auto-aggregation abilities of biofilm cells were associated with the higher surface adhesive forces but contradictory to lower basic surface charge and hydrophobicity than those of planktonic cells. The result suggested that the extracellular polymeric substance (EPS) attached to the surface of biofilm cells may mediate non-specific intercellular adhesion and further promote the intercellular adhesion of biofilm cells (Busscher et al., 2008). The strain specificity in the co-aggregation of *T. halophilus* with three pathogenic bacteria may be attributed to different intercellular molecular interactions. The co-aggregation of *T. halophilus* planktonic cells with *S. aureus* may be mainly driven by specific intercellular adhesion mediated by surface proteins such as lectins (Dufrêne and Viljoen, 2020), while a non-specific co-aggregation between *T. halophilus* biofilm cells and *S. Typhimurium* may be mediated by EPS. Negative values in the co-aggregation of LAB and *L. monocytogenes* were also reported previously (Garriga et al., 2015), and it may result from some antagonistic mechanisms that prevented co-aggregation and interfered auto-aggregation. The inhibitory effect of *T. halophilus* biofilms on the formation of pathogenic bacteria biofilms could be related to formation of acidic microenvironment or production of antimicrobial compounds. Taken together, *T. halophilus* can reduce the potential contamination of food products with pathogenic and spoilage microorganisms especially *S. aureus* and has potential probiotic properties.

## Conclusion

This study firstly investigated the biofilm formation by *T. halophilus* under various environment conditions and observed the biofilms by CLSM, AFM, and SEM. The results showed that *T. halophilus* preferred forming biofilm in a growable environment and on the surface of stainless steel. Further, we investigated the composition of biofilm, and proteins, polysaccharides, eDNA, and humic-like compounds were detected in biofilm. In addition, biofilm formation changed some physiological characters, increased the stress tolerance of cells, and inhibited the biofilm formation by some pathogenic bacteria. This work can serve as an open sesame to further analyses and applications of *T. halophilus* biofilms. Results presented in this study may contribute to our understanding of knowledge in biofilm formation by halophilic LABs and provide reference for study on bacterial biofilm.

## Data Availability Statement

The original contributions presented in the study are included in the article/supplementary material, further inquiries can be directed to the corresponding author/s.

## Author Contributions

SY carried out all experimental work, formal analysis, visualization, and writing of the original draft. LH contributed to data curation and formal analysis. RZ, YJ, and JH contributed to methodology. CW reviewed and edited the manuscript. All authors approved the final version of the manuscript and agreed to be accountable for all aspects of the work.

## Conflict of Interest

The authors declare that the research was conducted in the absence of any commercial or financial relationships that could be construed as a potential conflict of interest.

## Publisher’s Note

All claims expressed in this article are solely those of the authors and do not necessarily represent those of their affiliated organizations, or those of the publisher, the editors and the reviewers. Any product that may be evaluated in this article, or claim that may be made by its manufacturer, is not guaranteed or endorsed by the publisher.

## References

[B2] Akoğlu A. (2020). The effect of some environmental conditions on planktonic growth and biofilm formation by some lactic acid bacteria isolated from a local cheese in Turkey. *Biotechnol. Lett.* 42 481–492. 10.1007/s10529-020-02794-4 31927753

[B3] Al-Amshawee S. Yunus M. Y. B. M. Lynam J. G. Lee W. H. Dai F. Dakhil I. H. (2021). Roughness and wettability of biofilm carriers: a systematic review. *Environ. Technol. Inno.* 21:101233. 10.1016/j.eti.2020.101233

[B4] Bastard A. Coelho C. Briandet R. Canette A. Gougeon R. Alexandre H. (2016). Effect of biofilm formation by *Oenococcus oeni* on malolactic fermentation and the release of aromatic compounds in wine. *Front. microbiol.* 7:613. 10.3389/fmicb.2016.00613 27199942 PMC4846790

[B5] Bellenberg S. Huynh D. Poetsch A. Sand W. Vera M. (2019). Proteomics reveal enhanced oxidative stress responses and metabolic adaptation in *Acidithiobacillus ferrooxidans* biofilm cells on pyrite. *Front. Microbiol.* 10:592. 10.3389/fmicb.2019.00592 30984136 PMC6450195

[B6] Berlanga M. Guerrero R. (2016). Living together in biofilms: the microbial cell factory and its biotechnological implications. *Microb. Cell. Fact.* 15:165. 10.1186/s12934-016-0569-5 27716327 PMC5045575

[B7] Bonsaglia E. C. R. Silva N. C. C. Júnior A. F. Júnior J. P. A. Tsunemi M. H. Rall V. L. M. (2014). Production of biofilm by *Listeria monocytogenes* in different materials and temperatures. *Food Control* 35 386–391. 10.1016/j.foodcont.2013.07.023

[B8] Booth S. C. Workentine M. L. Wen J. Shaykhutdinov R. Vogel H. J. Ceri H. (2011). Differences in metabolism between the biofilm and planktonic response to metal stress. *J. Proteome Res.* 10 3190–3199. 10.1021/pr2002353 21561166

[B9] Busscher H. J. Norde W. van der Mei H. C. (2008). Specific molecular recognition and nonspecific contributions to bacterial interaction forces. *Appl. Environ. Microbiol.* 74 2559–2564. 10.1128/aem.02839-07 18344352 PMC2394898

[B10] Cai J. N. Jung J. E. Dang M. H. Kim M. A. Yi H. K. Jeon J. G. (2016). Functional relationship between sucrose and a cariogenic biofilm formation. *PLoS One* 11:e0157184. 10.1371/journal.pone.0157184 27275603 PMC4898727

[B11] Campana R. van Hemert S. Baffone W. (2017). Strain-specific probiotic properties of lactic acid bacteria and their interference with human intestinal pathogens invasion. *Gut Pathog.* 9:12. 10.1186/s13099-017-0162-4 28286570 PMC5338089

[B12] Campoccia D. Montanaro L. Arciola C. R. (2013). A review of the biomaterials technologies for infection-resistant surfaces. *Biomaterials* 34 8533–8554. 10.1016/j.biomaterials.2013.07.089 23953781

[B13] Casillo A. Lanzetta R. Parrilli M. Corsaro M. (2018). Exopolysaccharides from marine and marine extremophilic bacteria: structures, properties, ecological roles and applications. *Mar. Drugs* 16:69. 10.3390/md16020069 29461505 PMC5852497

[B14] Chaignon P. Sadovskaya I. Ragunah C. Ramasubbu N. Kaplan J. B. Jabbouri S. (2007). Susceptibility of staphylococcal biofilms to enzymatic treatments depends on their chemical composition. *Appl. Microbiol. Biotechnol.* 75 125–132. 10.1007/s00253-006-0790-y 17221196

[B15] Chamignon C. Guéneau V. Medina S. Deschamps J. Gil-Izquierdo A. Briandet R. (2020). Evaluation of the probiotic properties and the capacity to form biofilms of various *Lactobacillus strains*. *Microorganisms* 8:1053. 10.3390/microorganisms8071053 32679908 PMC7409210

[B16] Colloca M. E. Ahumada M. C. López M. E. Nader-Macías M. E. (2000). Surface properties of lactobacilli isolated from healthy subjects. *Oral. Dis.* 6 227–233. 10.1111/j.1601-0825.2000.tb00118.x 10918560

[B17] Costerton J. W. Stewart P. S. Greenberg E. P. (1999). Bacterial biofilms: a common cause of persistent infections. *Science* 284 1318–1322. 10.1126/science.284.5418.1318 10334980

[B18] Desai S. Sanghrajka K. Gajjar D. (2019). High adhesion and increased cell death contribute to strong biofilm formation in *Klebsiella pneumoniae*. *Pathogens* 8:277. 10.3390/pathogens8040277 31805671 PMC6963951

[B19] di Biase A. Kowalski M. S. Devlin T. R. Oleszkiewicz J. A. (2020). Physicochemical methods for biofilm removal allow for control of biofilm retention time in a high rate MBBR. *Environ. Technol.* [Epub ahead of print], 10.1080/09593330.2020.1843078 33161889

[B20] Di Bonaventura G. Piccolomini R. Paludi D. D’Orio V. Vergara A. Conter M. (2008). Influence of temperature on biofilm formation by Listeria monocytogenes on various food-contact surfaces: relationship with motility and cell surface hydrophobicity. *J. Appl. Microbiol.* 104 1552–1561. 10.1111/j.1365-2672.2007.03688.x 18194252

[B21] Dimakopoulou-Papazoglou D. Lianou A. Koutsoumanis K. P. (2016). Modelling biofilm formation of *Salmonella enterica* ser. newport as a function of pH and water activity. *Food Microbiol.* 53(Pt. B) 76–81. 10.1016/j.fm.2015.09.002 26678133

[B22] Dufrene Y. F. Persat A. (2020). Mechanomicrobiology: how bacteria sense and respond to forces. *Nat. Rev. Microbiol.* 18 227–240. 10.1038/s41579-019-0314-2 31959911

[B23] Dufrêne Y. F. Viljoen A. (2020). Binding strength of gram-positive bacterial adhesins. *Front. Microbiol.* 11:1457. 10.3389/fmicb.2020.01457 32670256 PMC7330015

[B24] Faille C. Bénézech T. Midelet-Bourdin G. Lequette Y. Clarisse M. Ronse G. (2014). Sporulation of *Bacillus* spp. within biofilms: a potential source of contamination in food processing environments. *Food Microbiol.* 40 64–74. 10.1016/j.fm.2013.12.004 24549199

[B25] Feldman-Salit A. Hering S. Messiha H. L. Veith N. Cojocaru V. Sieg A. (2013). Regulation of the activity of lactate dehydrogenases from four lactic acid bacteria. *J. Biol. Chem.* 288 21295–21306. 10.1074/jbc.M113.458265 23720742 PMC3774398

[B26] Flemming H.-C. Wingender J. (2010). The biofilm matrix. *Nat. Rev. Microbiol.* 8 623–633. 10.1038/nrmicro2415 20676145

[B27] Gambino M. Cappitelli F. (2016). Mini-review: biofilm responses to oxidative stress. *Biofouling* 32 167–178. 10.1080/08927014.2015.1134515 26901587

[B28] Gao L. Han F. Zhang X. Liu B. Fan D. Sun X. (2020). Simultaneous nitrate and dissolved organic matter removal from wastewater treatment plant effluent in a solid-phase denitrification biofilm reactor. *Bioresour. Technol.* 314:123714. 10.1016/j.biortech.2020.123714 32593786

[B29] García-Cayuela T. Korany A. M. Bustos I. de Cadiñanos L. P. G. Requena T. Peláez C. (2014). Adhesion abilities of dairy *Lactobacillus plantarum* strains showing an aggregation phenotype. *Food Res. Int.* 57 44–50. 10.1016/j.foodres.2014.01.010

[B30] Garriga M. Rubio R. Aymerich T. Ruas-Madiedo P. (2015). Potentially probiotic and bioprotective lactic acid bacteria starter cultures antagonise the *Listeria monocytogenes* adhesion to HT29 colonocyte-like cells. *Benef. Microbes* 6 337–343. 10.3920/bm2014.0056 25488261

[B31] Gloag E. S. Turnbull L. Huang A. Vallotton P. Wang H. Nolan L. M. (2013). Self-organization of bacterial biofilms is facilitated by extracellular DNA. *Proc. Natl. Acad. Sci. U.S.A.* 110 11541–11546. 10.1073/pnas.1218898110 23798445 PMC3710876

[B32] Han X. Zhang L. J. Wu H. Y. Wu Y. F. Zhao S. N. (2018). Investigation of microorganisms involved in kefir biofilm formation. *Anton. Leeuw.* 111 2361–2370. 10.1007/s10482-018-1125-6 30043188

[B33] Harada R. Yuzuki M. Ito K. Shiga K. Bamba T. Fukusaki E. (2018). Microbe participation in aroma production during soy sauce fermentation. *J. Biosci. Bioeng.* 125 688–694. 10.1016/j.jbiosc.2017.12.004 29366719

[B34] Hayrapetyan H. Muller L. Tempelaars M. Abee T. Nierop Groot M. (2015). Comparative analysis of biofilm formation by *Bacillus cereus* reference strains and undomesticated food isolates and the effect of free iron. *Int. J. Food Microbiol.* 200 72–79. 10.1016/j.ijfoodmicro.2015.02.005 25700364

[B35] Izano E. A. Amarante M. A. Kher W. B. Kaplan J. B. (2008). Differential roles of poly-N-acetylglucosamine surface polysaccharide and extracellular DNA in *Staphylococcus aureus* and *Staphylococcus epidermidis* biofilms. *Appl. Environ. Microbiol.* 74 470–476. 10.1128/aem.02073-07 18039822 PMC2223269

[B36] Jakubovics N. S. Shields R. C. Rajarajan N. Burgess J. G. (2013). Life after death: the critical role of extracellular DNA in microbial biofilms. *Lett. Appl. Microbiol.* 57 467–475. 10.1111/lam.12134 23848166

[B37] Jefferson K. K. (2004). What drives bacteria to produce a biofilm? *FEMS Microbiol. Lett.* 236 163–173. 10.1016/j.femsle.2004.06.005 15251193

[B38] Jeong D. W. Heo S. Lee J. H. (2017). Safety assessment of *Tetragenococcus halophilus* isolates from doenjang, a korean high-salt-fermented soybean paste. *Food Microbiol.* 62 92–98. 10.1016/j.fm.2016.10.012 27889172

[B39] Jingjing E. Rongze M. Zichao C. Caiqing Y. Ruixue W. Qiaoling Z. (2021). Improving the freeze-drying survival rate of *Lactobacillus plantarum* LIP-1 by increasing biofilm formation based on adjusting the composition of buffer salts in medium. *Food Chem.* 338:128134. 10.1016/j.foodchem.2020.128134 33091996

[B40] Kalaiarasan E. Thirumalaswamy K. Harish B. N. Gnanasambandam V. Sali V. K. John J. (2017). Inhibition of quorum sensing-controlled biofilm formation in *Pseudomonas aeruginosa* by quorum-sensing inhibitors. *Microb. Pathog.* 111 99–107. 10.1016/j.micpath.2017.08.017 28818490

[B41] Kim S.-H. Park C. Lee E.-J. Bang W.-S. Kim Y.-J. Kim J.-S. (2017). Biofilm formation of campylobacter strains isolated from raw chickens and its reduction with DNase I treatment. *Food Control* 71 94–100. 10.1016/j.foodcont.2016.06.038

[B42] Kragh K. N. Hutchison J. B. Melaugh G. Rodesney C. Roberts A. E. Irie Y. (2016). Role of multicellular aggregates in biofilm formation. *mBio* 7:e00237. 10.1128/mBio.00237-16 27006463 PMC4807362

[B43] Kruger N. J. (1994). The bradford method for protein quantitation. *Protein Prot. Handbook Springer* 32 9–15. 10.1385/0-89603-268-x:9 7951753

[B44] Kubota H. Senda S. Nomura N. Tokuda H. Uchiyama H. (2008). Biofilm formation by lactic acid bacteria and resistance to environmental stress. *J. Biosci. Bioeng.* 106 381–386. 10.1263/jbb.106.381 19000615

[B45] León-Romero Á Domínguez-Manzano J. Garrido-Fernández A. Arroyo-López F. N. Jiménez-Díaz R. (2016). Formation of in vitro mixed-species biofilms by *Lactobacillus pentosus* and yeasts isolated from spanish-style green table olive fermentations. *Appl. Environ. Microbiol.* 82 689–695. 10.1128/aem.02727-15 26567305 PMC4711112

[B46] Li D. Liang X. Wu C. (2020). Characteristics of nitrogen removal and extracellular polymeric substances of a novel salt-tolerant denitrifying bacterium, *Pseudomonas* sp. DN-23. *Front. Microbiol.* 11:335. 10.3389/fmicb.2020.00335 32210936 PMC7067702

[B47] Ma L. Jackson K. D. Landry R. M. Parsek M. R. Wozniak D. J. (2006). Analysis of *Pseudomonas aeruginosa* conditional psl variants reveals roles for the psl polysaccharide in adhesion and maintaining biofilm structure postattachment. *J. Bacteriol.* 188 8213–8221. 10.1128/jb.01202-06 16980452 PMC1698210

[B48] Marsden A. E. Grudzinski K. Ondrey J. M. DeLoney-Marino C. R. Visick K. L. (2017). Impact of salt and nutrient content on biofilm formation by *Vibrio fischeri*. *PLoS One* 12:e0169521. 10.1371/journal.pone.0169521 28122010 PMC5266276

[B49] Mieher J. L. Larson M. R. Schormann N. Purushotham S. Wu R. Rajashankar K. R. (2018). Glucan binding protein C of *Streptococcus mutans* mediates both sucrose-independent and sucrose-dependent adherence. *Infect. Immun.* 86:e00146-18. 10.1128/iai.00146-18 29685986 PMC6013656

[B50] Parsek M. R. Singh P. K. (2003). Bacterial biofilms: an emerging link to disease pathogenesis. *Annu. Rev. Microbiol.* 57 677–701. 10.1146/annurev.micro.57.030502.090720 14527295

[B51] Philips J. Rabaey K. Lovley D. R. Vargas M. (2017). Biofilm formation by *Clostridium ljungdahlii* is induced by sodium chloride stress: experimental evaluation and transcriptome analysis. *PLoS One* 12:e0170406. 10.1371/journal.pone.0170406 28118386 PMC5261816

[B52] Piwat S. Sophatha B. Teanpaisan R. (2015). An assessment of adhesion, aggregation and surface charges of *Lactobacillus strains* derived from the human oral cavity. *Lett. Appl. Microbiol.* 61 98–105. 10.1111/lam.12434 25913304

[B53] Prechtl R. M. Janßen D. Behr J. Ludwig C. Küster B. Vogel R. F. (2018). Sucrose-induced proteomic response and carbohydrate utilization of *Lactobacillus sakei* TMW 1.411 during dextran formation. *Front. Microbiol.* 9:2796. 10.3389/fmicb.2018.02796 30532743 PMC6265474

[B54] Pumeesat P. Muangkaew W. Ampawong S. Luplertlop N. (2017). Candida albicans biofilm development under increased temperature. *New Microbiol.* 40 279–283. 28825445

[B55] Ribeiro S. M. Felício M. R. Boas E. V. Gonçalves S. Costa F. F. Samy R. P. (2016). New frontiers for anti-biofilm drug development. *Pharmacol. Ther.* 160 133–144. 10.1016/j.pharmthera.2016.02.006 26896562

[B56] Sager M. Benten W. P. Engelhardt E. Gougoula C. Benga L. (2015). Characterization of biofilm formation in [pasteurella] pneumotropica and [actinobacillus] muris isolates of mouse origin. *PLoS One* 10:e0138778. 10.1371/journal.pone.0138778 26430880 PMC4592018

[B57] Schlegelová J. Karpísková S. (2007). [Microbial biofilms in the food industry]. *Epidemiol. Mikrobiol. Imunol.* 56 14–19. 17427749

[B58] Slížová M. Nemcová R. Mad’ar M. Hadryová J. Gancarčíková S. Popper M. (2015). Analysis of biofilm formation by intestinal lactobacilli. *Can. J. Microbiol.* 61 437–446. 10.1139/cjm-2015-0007 25961850

[B59] Souza J. G. S. Cury J. A. Ricomini Filho A. P. Feres M. Faveri M. Barão V. A. R. (2019). Effect of sucrose on biofilm formed in situ on titanium material. *J. Periodontol.* 90 141–148. 10.1002/jper.18-0219 30070706

[B60] Speranza B. Corbo M. R. Campaniello D. Altieri C. Sinigaglia M. Bevilacqua A. (2020). Biofilm formation by potentially probiotic *Saccharomyces cerevisiae* strains. *Food Microbiol.* 87:103393. 10.1016/j.fm.2019.103393 31948634

[B61] Stewart P. S. Franklin M. J. (2008). Physiological heterogeneity in biofilms. *Nat. Rev. Microbiol.* 6 199–210. 10.1038/nrmicro1838 18264116

[B62] Tango C. N. Akkermans S. Hussain M. S. Khan I. Van Impe J. Jin Y. G. (2018). Modeling the effect of pH, water activity, and ethanol concentration on biofilm formation of *Staphylococcus aureus*. *Food Microbiol.* 76 287–295. 10.1016/j.fm.2018.06.006 30166152

[B63] Taniguchi M. Hoshino K. Shimizu K. Nakagawa I. Takahashi Y. Fujii M. (1988). Rapid production of *Pediococcus halophilus* salt-tolerant cells by a cultivation method employing gradual increases in NaCl concentration using a fermentor with a microfiltration module. *J. Ferment. Technol.* 66 633–641.

[B64] Tarrah A. da Silva Duarte V. de Castilhos J. Pakroo S. Junior W. J. F. L. Luchese R. H. (2019). Probiotic potential and biofilm inhibitory activity of *Lactobacillus casei* group strains isolated from infant feces. *J. Funct. Foods* 54 489–497. 10.1016/j.jff.2019.02.004

[B65] Tatsaporn T. Kornkanok K. (2020). Using potential lactic acid bacteria biofilms and their compounds to control biofilms of foodborne pathogens. *Biotechnol. Rep.* 26:e00477. 10.1016/j.btre.2020.e00477 32509542 PMC7264490

[B66] Teng M. Yu D. Cao B. Wang D. Zhang W. (2016). Variations in distribution and composition of extracellular polymeric substance (EPS) of biological sludge under potassium ferrate conditioning: effects of pH and ferrate dosago. *Biochem. Eng. J.* 106 37–47.

[B67] Thibeaux R. Soupé-Gilbert M. E. Kainiu M. Girault D. Bierque E. Fernandes J. (2020). The zoonotic pathogen *Leptospira interrogans* mitigates environmental stress through cyclic-di-GMP-controlled biofilm production. *NPJ Biofilms Microbi.* 6:24. 10.1038/s41522-020-0134-1 32532998 PMC7293261

[B68] Udomsil N. Chen S. Rodtong S. Yongsawatdigul J. (2017). Improvement of fish sauce quality by combined inoculation of *Tetragenococcus halophilus* MS33 and virgibacillus sp. SK37. *Food Control* 73 930–938. 10.1016/j.foodcont.2016.10.007

[B69] Van Houdt R. Michiels C. W. (2010). Biofilm formation and the food industry, a focus on the bacterial outer surface. *J. Appl. Microbiol.* 109 1117–1131. 10.1111/j.1365-2672.2010.04756.x 20522145

[B70] Volle C. B. Ferguson M. A. Aidala K. E. Spain E. M. Núñez M. E. (2008). Spring constants and adhesive properties of native bacterial biofilm cells measured by atomic force microscopy. *Colloids Surface B* 67 32–40. 10.1016/j.colsurfb.2008.07.021 18815013

[B71] Wang H. Wu N. Jiang Y. J. Ye K. Xu X. Zhou G. (2016). Response of long-term acid stress to biofilm formation of meat-related *Salmonella* Enteritidis. *Front. Microbiol.* 69:214–220.

[B72] Wang M. Liu Q. Kang X. Zhu Z. Yang H. Xi X. (2020). Glycogen metabolism impairment via single gene mutation in the glgBXCAP operon alters the survival rate of *Escherichia coli* under various environmental stresses. *Front. Microbiol.* 11:588099. 10.3389/fmicb.2020.588099 33101261 PMC7546213

[B73] Welman A. D. Maddox I. S. (2003). Exopolysaccharides from lactic acid bacteria: perspectives and challenges. *Trends Biotechnol.* 21 269–274. 10.1016/s0167-7799(03)00107-0 12788547

[B74] Wu C. Liu C. He G. Huang J. Zhou R. (2013). Characterization of a multiple-stress tolerance *Tetragenococcus halophilus* and application as starter culture in Chinese horsebean-chili-paste manufacture for quality improvement. *Food Sci. Technol. Res.* 19 855–864. 10.3136/fstr.19.855

[B75] Xiong Y. Liu Y. (2013). Importance of extracellular proteins in maintaining structural integrity of aerobic granules. *Colloid Surface B* 112 435–440. 10.1016/j.colsurfb.2013.07.060 24036627

[B76] Yao S. Hao L. Zhou R. Jin Y. Huang J. Wu C. (2021). Co-culture with *Tetragenococcus halophilus* improved the ethanol tolerance of *Zygosaccharomyces rouxii* by maintaining cell surface properties. *Food Microbiol.* 97:103750. 10.1016/j.fm.2021.103750 33653523

[B77] Zhang B. Yang X. Liu L. Chen L. Teng J. Zhu X. (2021). Spatial and seasonal variations in biofilm formation on microplastics in coastal waters. *Sci. Total. Environ.* 770:145303. 10.1016/j.scitotenv.2021.145303 33515883

[B78] Zhang G. Lu M. Liu R. Tian Y. Vu V. H. Li Y. (2020). Inhibition of *Streptococcus mutans* biofilm formation and virulence by *Lactobacillus plantarum* K41 isolated from traditional sichuan pickles. *Front. Microbiol.* 11:774. 10.3389/fmicb.2020.00774 32425911 PMC7203412

[B79] Zheng D. Gao M. Wang Z. She Z. Jin C. Chang Q. (2016). Performance comparison of biofilm and suspended sludge from a sequencing batch biofilm reactor treating mariculture wastewater under oxytetracycline stress. *Environ. Technol.* 37 2391–2404. 10.1080/09593330.2016.1150353 26854088

